# Philadelphia College of Dental Surgery

**Published:** 1852-07

**Authors:** 


*Philadelphia College of Dental Surgery.*—In acknowledging the receipt of the first annual announcement of the Philadelphia College of Dental Surgery, we tender the faculty the right hand of fellowship. We welcome them to a field of labor in which we, for some years, have been toiling. We do this, because we have the fullest confidence in the ability of the gentlemen to discharge the responsible duties they have severally assumed in this institution, and most sincerely do we wish them success in the noble enterprize in which they are engaged.

The names of the gentlemen filling the several chairs, we announced in a former number of the Journal. The lectures, commencing the first Monday in November, will continue four months. The means of instruction, which the Professors will be able to command, will secure to the students advantages which can no where be obtained from private tuition.

